# Contribution of cell death signaling to blood vessel formation

**DOI:** 10.1007/s00018-020-03738-x

**Published:** 2021-03-30

**Authors:** Nathalie Tisch, Carmen Ruiz de Almodóvar

**Affiliations:** Department of Vascular Dysfunction, European Center for Angioscience (ECAS), Faculty of Medicine Mannheim, University of Heidelberg, Mannheim, Germany

**Keywords:** Vessel pruning, Angiogenesis, Endothelial cells, Caspases, Apoptosis

## Abstract

The formation of new blood vessels is driven by proliferation of endothelial cells (ECs), elongation of maturing vessel sprouts and ultimately vessel remodeling to create a hierarchically structured vascular system. Vessel regression is an essential process to remove redundant vessel branches in order to adapt the final vessel density to the demands of the surrounding tissue. How exactly vessel regression occurs and whether and to which extent cell death contributes to this process has been in the focus of several studies within the last decade. On top, recent findings challenge our simplistic view of the cell death signaling machinery as a sole executer of cellular demise, as emerging evidences suggest that some of the classic cell death regulators even promote blood vessel formation. This review summarizes our current knowledge on the role of the cell death signaling machinery with a focus on the apoptosis and necroptosis signaling pathways during blood vessel formation in development and pathology.

## Introduction

Blood vessels are lumenized structures with an inner lining of a single layer of endothelial cells (ECs) that transport oxygen and nutrients throughout the body. After the de-novo formation of an initial primitive vascular system by accumulation of angioblasts and blood islands (vasculogenesis), the vascular system develops by sprouting of new vessels from already pre-existing ones (angiogenesis) [1]. Angiogenesis is followed by EC differentiation into arteries, veins and capillaries and ultimately vessel remodeling to acquire a hierarchically structured vascular system. The diversity of these processes indicates that factors regulating EC survival must be balanced to allow resistance to exogenous stresses during organ growth, while promoting vessel pruning (removal of single vessel segments during the process of vascular optimization) or regression (defined here as elimination of multiple vessel segments as part of a mechanism of vascular rarefaction) and thus maturation of the vascular system. Even though the adult vascular system is very stable, with a low turnover of ECs during homeostatic conditions, the endothelium maintains its plastic capacity. For example, the vasculature readily adapts to the metabolic demands of its surrounding tissue by either reducing or increasing vessel density, such as in physiological conditions like the regression of the corpus luteum during the female reproductive cycle [2], exercise [3, 4] or wound healing [5], or in pathological conditions like malignant tumor growth [6].

Whereas it is undoubted that EC death contributes to the complete removal of certain vascular networks during development, such as the hyaloid vasculature of the fetal retina [7–9] or the first, second and fifth aortic arches [10, 11] during the formation of the cardiovascular system, it seems that cell death is not a key driver for later stages of vessel pruning, when vessel branches only partially regress to improve the functionality of an existing vascular network [12]. Recent evidences suggest that under such conditions, the activity of the cell death signaling machinery can even promote angiogenesis and blood vessel development [13–15].

In addition to these physiological examples, excessive EC death can also lead to pathological vessel regression, such as it occurs during retinopathy of prematurity (ROP), when preterm infants are exposed to high oxygen levels. Vice versa, impaired EC death can cause persistent hyperplastic primary vitreous, a developmental human eye disease caused by defective hyaloid vessel regression [16, 17]. On the other hand, targeting the cell death signaling machinery in ECs to inhibit blood vessel formation and vascularization during cancer development, thus promoting tumor regression, could be used as a valuable therapeutical strategy [18].

**Fig. 1:**
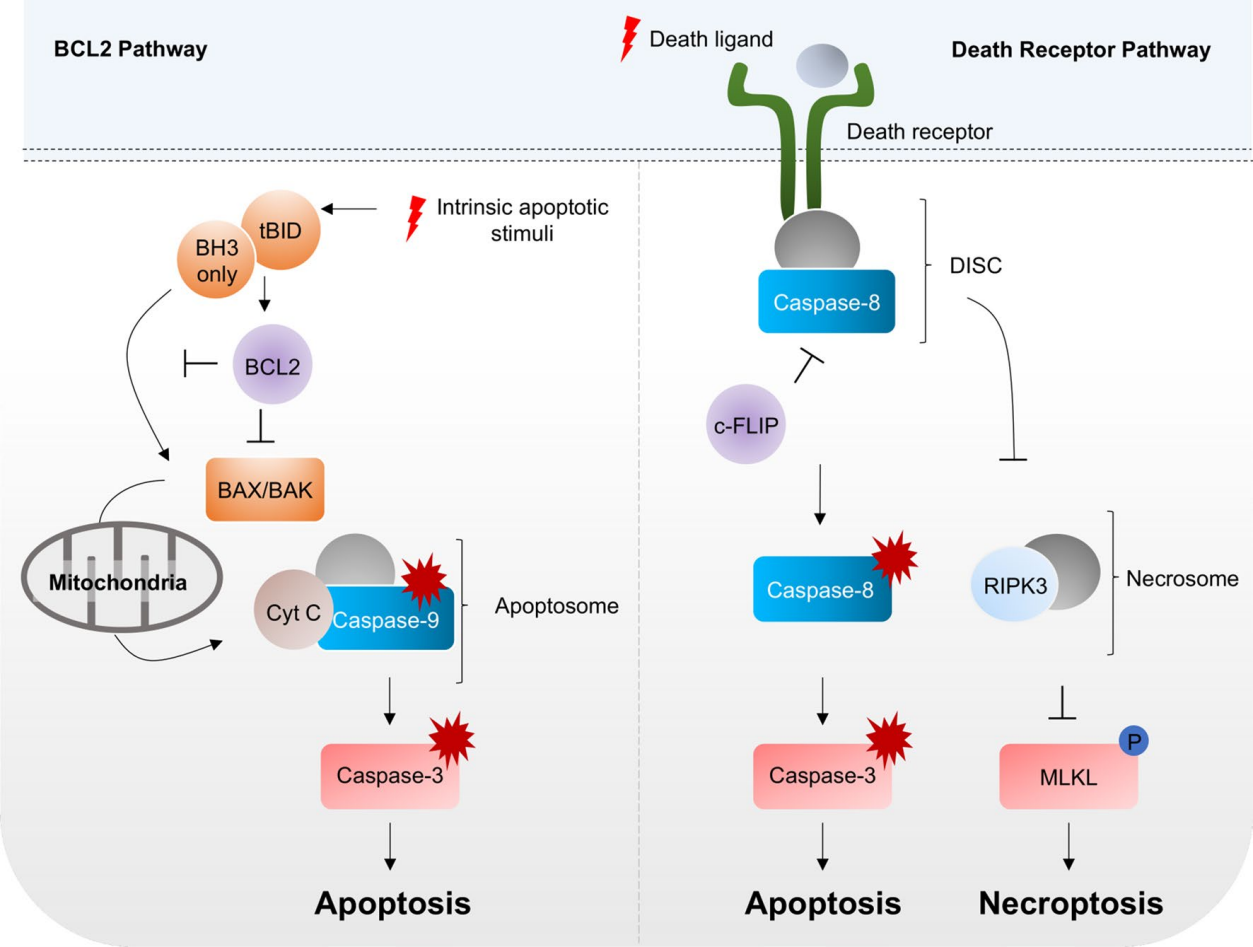
**Simplified schematic overview of the BCL2-regulated and death receptor-mediated apoptosis and necroptosis signaling pathways**. In the BCL2 pathway, interactions between pro- and anti-apoptotic BCL2 sub-family members determine whether the pro-apoptotic effectors BAK and BAX become activated. This results in increased mitochondrial permeability and cytochrome C (Cyt C) release, which, in turn, favors the formation of the death inducing apoptosome and ultimately Caspase-3 (CASP-3) cleavage by the initiator Caspase-9 (CASP-9). In the extrinsic pathway, binding of a death ligand to its death receptor leads to formation of the death inducing signaling complex (DISC) and activation of the initiator Caspase-8 (CASP-8). In the DISC, CASP-8 activity is restricted when CASP-8 binds to its inhibitor c-FLIP. Full CASP-8 activity in the absence of c-FLIP leads to CASP-3 cleavage, activation, and cell death by apoptosis. On the other hand, a minimal CASP-8 activity within the DISC is required to inhibit cell death via necroptosis. Loss of CASP-8 or inhibition of its enzymatic activity result in the formation of the necrosome including RIPK3, and ultimately activation and phosphorylation of the necroptosis executer MLKL. For reasons of simplicity, only key molecules of the different signaling complexes are highlighted. For further reading, we kindly refer the reader to reference [19]

We start this review with a brief introduction into the different cell death signaling pathways regulating apoptosis and necroptosis (Fig. 1) and discuss how these are required to promote full regression of specific vascular systems, using the hyaloid vasculature of the fetal retina as an example. We will then summarize our current knowledge about vessel remodeling at later developmental stages and highlight the cell death independent functions of the cell death signaling machinery to this process. Finally, we will discuss the pathological contributions of EC death to vascular diseases, and its benefits in anti-angiogenic cancer therapy.

## Molecular regulation of apoptosis and necroptosis

Two separated, but converging, pathways control apoptosis (Fig. 1): one is the so-called intrinsic, mitochondria dependent pathway regulated by the BCL2 family of proteins and the other one is regulated via extrinsic factors that activate the death receptor induced cell death signaling pathway. Both pathways result in the activation of initiator Caspases such as Caspase-8 (CASP-8) and -9 that in turn cleave and activate downstream Caspases (e.g. CASP-3 and -7) to implement the cell death program [19].

### Intrinsic cell death signaling pathway

Proteins of the BCL2 family share the presence of short segments of sequence homology referred to as the BCL2 homology (BH) regions and are divided into three sub-families that can have pro- or anti-apoptotic properties [20]. BCL2 has been the first identified member of the pro-survival sub-family, which also includes BCLxL, MCL1, and others [20, 21]. The other two sub-families are pro-apoptotic. The BH3-only sub-family (e.g. BIM, BID, BAD, PUMA), so-named because its members only share the BH3-region [22], are regulated by apoptotic stimuli and initiate apoptosis via BAX and BAK, members of the other pro-apoptotic sub-family [23]. Upon oligomerization, BAX and BAK are responsible for mitochondrial outer membrane permeabilization (MOMP) [24, 25]. Interactions between the three BCL2 sub-families determine whether BAX and BAK become activated, as pro-survival BCL2 family proteins can either prevent BH3-only proteins from binding and thus activating BAX and BAK [26] or directly bind to the activated forms of BAX and BAK [27]. Recently, BOX was identified as a non-canonical pro-apoptotic BCL2 family member [28]. Whereas 10% of *BAK/BAX* double knockout mice survive to adulthood, with only minor developmental defects, the phenotype of *BOX/BAK/BAX* triple knockout mice is much more severe, with less than 2% of the mice surviving to adulthood [29]. While these findings demonstrate a certain redundancy amongst the pro-apoptotic BCL2 family members, it also shows that life in the absence of intrinsic apoptosis is possible.

During cell death, MOMP induces the release of Cytochrome C (Cyt C) [25] and other pro-apoptotic molecules. In turn, Cyt C in the cytoplasm favors the formation of the so-called ‘apoptosome’, a signaling platform that recruits and activates the initiator CASP-9 [30] which will then further cleave and activate the executioner CASP-3. Executioner Caspases have the ability to cleave diverse cellular substrates, such as lamin-β1 [31], β-actin [32] and poly (ADP-ribose) polymerase [33] and thus to induce the regulated cellular degradation during apoptosis. Not only growth factor deprivation, but also other intracellular risk factors such as DNA damage, hypoxia, or metabolic stress can activate cell death via the mitochondria-mediated intrinsic apoptosis pathway [34–36].

### Extrinsic cell death signaling pathway and necroptosis

The extrinsic cell death signaling pathway is exogenously induced at a cell’s plasma membrane when a death ligand binds to its respective death receptor. Eight members of the death receptor family have been described so far, with the tumor necrosis factor (TNF) receptor superfamily, including TNFR1, TRAILR2 and CD95, being the most important ones [37]. All of them are type 1 transmembrane receptors that can be distinguished by a ~ 80 amino acids comprising cytoplasmic domain, known as the death domain (DD). Signal transduction of death receptors takes place in three general steps: 1) Binding of the death ligand to its receptor; 2) recruitment of adaptor proteins and the initiator CASP-8 (in humans CASP-10 is also recruited) to the DD, resulting in the formation of the death inducing signaling complex (DISC); 3) specific downstream signaling events depending on the stoichiometry of adaptor proteins and initiator caspases. Large amounts of activated initiator caspases can directly process and activate the executioner CASP-3, CASP-6 or CASP-7 [38]. However, in the presence of caspase inhibitory molecules of the inhibitor of apoptosis (IAP) protein family, or if smaller amounts of activated CASP-8 are available, efficient apoptosis depends on cleavage of the BH3-only protein BID by CASP-8. Truncated BID (tBID) can then translocate to the mitochondria to induce oligomerization of BAK and BAX to amplify cell death via the intrinsic pathway [38, 39].

CASP-8 activity is normally restricted and inhibited by heterodimerization with c-FLIP, a homologous protein that lacks catalytic activity [40]. Even though c-FLIP prevents the full activation of CASP-8, a restricted enzymatic activity of CASP-8 within the c-FLIP/CASP-8 heterodimer is necessary to prevent an alternative cell death pathway called necroptosis [41]. Necroptosis is induced upon autophosphorylation and activation of the receptor interacting protein kinases (RIPK) 1 and 3 [42, 43]. CASP-8 can directly cleave RIPK1 and RIPK3 [44, 45] and, therefore, prevent their kinase activity. In addition, CASP-8 indirectly inhibits RIPK1 by cleaving the deubiquitinase cylindromatosis (CYLD), as ubiquitinated RIPK1 cannot be recruited to the cytosolic death signaling complex anymore [46]. Mixed lineage kinase domain like protein (MLKL) is a target of RIPK1 and RIPK3 and the key executor of necroptosis [47, 48]. Phosphorylation of MLKL by RIPK3 induces a conformational change that promotes MLKL oligomerization and translocation to the plasma membrane. It is hypothesized that tetrameric MLKL can integrate into the plasma membrane, induce pore formation and thus cause the classical cellular rupture seen in necrotic cells [49, 50].

## Regulation of cell death during development of the embryonic vascular system

Formation of the cardiovascular system starts around embryonic day (E)8 in mice [51]. Soon after the formation of the heart rudiments and looping and formation of the heart chambers, intraembryonic blood vessels start to form independently by accumulation of angioblasts that will later on connect to the heart circulation [52]. Whereas it is well described that some of the primordial pharyngeal arch arteries are removed by apoptosis  in the developing mammalian heart [53], only little is known about the contribution of cell death in ECs of the developing embryonic microvasculature.

Studies using genetic mouse models (Table 1) suggest that the embryonic vasculature is highly sensitive to EC death. Increased EC apoptosis often goes in hand with hemorrhage formation, defects in yolk sac vascularization and embryonic lethality. TAK1 is a kinase induced downstream of the TNFR1, where it activates Nf-κB signaling upon TNF-α stimulation [54]. EC-specific deletion of *Tak1* leads to increased TNF-dependent EC apoptosis, reduced embryo angiogenesis, defects in yolk sac vascularization and ultimately embryonic lethality around E10.5 [55]. Other molecules of the extrinsic cell death pathway have also been implicated in regulating EC survival during embryonic development. For example, *cFlip*, *Casp8,* and *Fadd* knockout mice die during early embryonic development between E10.5–12.5 due to cardiovascular failure and compromised yolk sac vascularization [56–58]. These defects are rescued when either apoptosis or necroptosis are inhibited, as shown in double or triple knockout mice like *c-FLIP*^*ko*^*/FADD*^*ko*^*/RIPK3*^*ko*^ [59], *Casp8*^*ko*^*/RIPK3*^*ko*^ [60] or *Casp8/MLKL*^*ko*^ [61]* mice.* In particular, EC-specific deletion of *Casp8* [13, 62], *FADD* [63] and other pro-survival molecules [55, 64] of the extrinsic cell death signaling pathway recapitulates the severity of the full knockout of these genes, indicating that pro-survival molecules of the extrinsic cell death signaling pathway are required to promote EC survival during embryonic development. Additionally, a pro-survival role for the heterodimer of CASP-8 and c-FLIP was described in multiple other tissues in vivo [59].

**Table 1:** Overview of the mouse mutants discussed in this review

Mouse line		Vascular phenotype			Reference
Name	Description	Embryonic development	Postnatal angiogenesis	Pathology	
c-FLIP^-/-^	Ubiquitous knockout of the anti-apoptotic c-FLIP	Impaired cardiovascular development, embryonic lethality at ~ E10.5, defective yolk sac vascularization	n.d.	n.d.	Yeh et al. (2000)
Casp8^-/-^	Ubiquitous knockout of the initiator CASP-8	Impaired cardiovascular development, embryonic lethality a ~ E10.5, defective yolk sac vascularization	n.d.	n.d.	Varfolomeev et al. (1998)
Casp-8^ECko^	Endothelial cell specific knockout of the initiator CASP-8	Hemorrhages, embryonic lethality at E13.5, defective yolk sac vascularization	Impaired retina angiogenesis	OIR model: Increased vaso-obliteration, reduced tuft formation. B16 melanoma metastasis model: Increased metastatic spread	Kang et al. (2004); Tisch et al. (2019); Strilic et al. (2016)
FADD^-/-^	Ubiquitous knockout of the adaptor protein FADD	Impaired cardiovascular development, embryonic lethality at ~ E10.5, defective yolk sac vascularization	n.d.	n.d.	Yeh et al. (1998)
FADD^ECko^	Endothelial cell specific knockout of the adaptor protein FADD	Impaired cardiovascular development, embryonic lethality at E11.5	n.d.	n.d.	Fan et al. (2016)
BAX^-/-^/BAK^-/-^	Ubiquitous knockout of the proapoptotic BCl2 members BAX and BAK	Improper formation of the aortic arches (phenotype not 100% penetrant)	Reduced hyaloid vessel regression	n.d.	Ke et al. (2018); Hahn et al. (2005)
BOX^-/-^/BAX^-/-^/BAK^-/-^	Ubiquitous knockout of the proapoptotic BCl2 members BOX, BAX and BAK	Less than 2% of the triple knockouts survive into adulthood	n.d.	n.d.	Ke et al. (2018)
PU.1^-/-^	Mouse model lacking macrophages	Reduced vascular anastomosis and vascular complexity in the embryonic hindbrain	Reduced hyaloid vessel regression	n.d.	Fantin et al. (2010); Lobov et al. (2005)
BIM^-/-^	Ubiquitous knockout of the proapoptotic BCL2 member BIM	n.d.	Reduced hyaloid vessel regression	Protection from hyperoxia induced vaso-obliteration	Wang et al. (2011); König et al. (2014)
BIM^PC^	Pericyte specific knockout of the proapoptotic BCL2 member BIM	n.d.	Increased pericyte and EC numbers, decreased apoptosis and proliferation of vascular associated cells, reduced hyaloid vessel regression, enhanced vertical sprouting into the deep vascular plexus	No protection from hyperoxia induced vaso-obliteration	Wang et al. (2017)
BIM^EC^	Endothelial cell specific knockout of the proapoptotic BCL2 member BIM	n.d.	Increased EC numbers, decreased apoptosis and proliferation of vascular associated cells, reduced hyaloid vessel regression, enhanced vertical sprouting into the deep vascular plexus	No protection from hyperoxia induced vaso-obliteration	Wang et al. (2017)
BIK^-/-^	Ubiquitous knockout of the proapoptotic BCL2 member BIK	No reported vascular abnormalities	Normal hyaloid vessel regression	n.d.	Coultas et al. (2004)
Endosialin^-/-^	Ubiquitous knockout of the type I transmembrane glycoprotein Endosialin which is expressed by pericytes	n.d.	Increased vessel density, reduced vessel regression	B16-xenograft: Reduced vessel remodeling in the primary tumor	Simonavicious et al. (2012)
Tie-1^ECko^	Endothelial cell specific knockout of theTIE1 receptor	n.d.	Increased EC death, reduced vessel density in the retina	B16-xenograft: Reduced tumor angiogenesis, increased tumor EC apoptosis	Savant et al. (2015)
Evi^ECko^	Endothelial cell specific knockout of the wnt secretion factor Evi	n.d.	Increased EC death, reduced vessel density in the retina, reduced EC proliferation, downregulation of survival genes	LLC model: reduced tumor vessel density	Korn et al. (2014)
RSPO3^ECko^	Endothelial cell specific knockout of the wnt signaling enhancer R-spondin-3	Embryonic lethality between E10.5–E13.5, defective yolk sac vascularization	Reduced retina vessel density, increased EC apoptosis	LLC model: reduced tumor vessel density, increased vessel regression	Scholz et al. (2016)
Mcl1^ECko^	Endothelial cell specific knockout of the anti-apoptotic BCL2 member Mcl1	Edema, hemorrhages, abnormal angiogenesis in the dorsal skin, embryonic lethality at E14.5	Reduced retina vessel density, increased EC apoptosis in proliferative areas, no defect in vessel regression	n.d.	Watson et al. (2016)
BAX^-/-^ BAK^EC/EC^	Ubiquitous knockout of the proapoptotic BCL2 member BAX combined with the endothelial cell specific knockout of BAK	n.d.	Reduced hyaloid vessel regression, reduced retina EC apoptosis, but no difference in vessel regression and vessel density; increased capillary EC density	OIR model: No EC apoptosis during vaso-obliteration phase. However, vessel regression still occurs. Increased revascularization upon return to normoxia	Watson et al. (2016); Grant et al. (2020)
TAK1^ECko^	Endothelial cell specific knockout of the prosurvival protein TAK1	Embryonic lethalty at E10.5–E11.5, reduced angiogenesis, disorganized vasculature, defective yolk sac vascu-larization, increased TNF dependent EC apoptosis	n.d.	EL4 lymphoma and LLC model: Reduced tumor vessel density, increased tumor vessel permeability	Morioka et al. (2012); Naito et al. (2019)
Survivin^ECko^	Endothelial cell specific knockout of the IAP protein survivin	Diffuse hemorrhages, abnormal heart development, increased EC apoptosis, embryonic lethality at E13.5, defective yolk sac vascularization	n.d.	n.d.	Zwerts et al. (2007)
PUMA^-/-^	Ubiquitous knockout of the proapoptotic BCL2 member PUMA	n.d.	Reduced vessel density and EC number retina and brain. No difference in apoptosis	CNV: Reduced choroidal neovascularization	Zhang et al. (2012)
CD95^ECko^	Endothelial cell specific knockout of the death receptor CD95	n.d.	Reduced vessel density in the retina and brain, reduced EC proliferation, no difference in vessel regression	n.d.	Chen et al. (2017)
TRAIL^-/-^	Ubiquitous knockout of the death ligand TRAIL	n.d.	Increased vessel density in the retina at P3, but not P5 and later; small but significant decrease in the capillary free area surrounding the arteries	OIR: No difference in vaso-obliteration; increased number and delayed regression of neovascular tufts due to decreased EC apoptosis	Hubert et al. (2009)
Fasl^gld/gld^	Loss of function mutation of the death ligand FasL (CD95L)	n.d.	n.d.	OIR: No difference in vaso-obliteration, increased formation of neovascular tufts, reduced EC apoptosis	Barreiro et al. (2003); Davies et al. (2003)
Bcl-2^-/-^	Ubiquitous knockout of the anti-apoptotic BCL2 member Bcl2	No reported vascular abnormalities	No difference in hyaloid vessel regression, decreased vessel density, decreased EC and PC numbers, increased apoptosis in retinal vessels	No protection from hyperoxia induced vaso-obliteration, reduced tuft formation	Wang et al. (2005)
Bcl-2^ECko^	Endothelial cell specific knockout of the anti-apoptotic BCL2 member Bcl2	n.d.	Reduced EC numbers at P21 and P42, decreased retinal artery and vein number, no difference in vessel outgrowth, increased apoptosis and proliferation at P14	OIR: Increased vaso-obliteration, no difference in pathological tuft formation. CNV: Reduced neovascularization	Zaitoun et al. (2015)

*n.d. (not determined)* vasculature at a specific developmental stage or pathology has not been examined in detail

Interestingly, *Tnfr1*, *TRAILR2* and *CD95* knockout mice are viable and develop without obvious impairments [65–67], suggesting that death receptor signaling (the extrinsic cell death signaling pathway) in ECs is not essential for the proper formation of the embryonic vascular system. Nevertheless, it is possible that these mice still present transient vascular defects that are compensated during development and therefore do not result in a permanent vascular defect. For example, vessel density and complexity are attenuated in EC-specific *CD95* knockout embryos [14].

Whereas the deathly potential of the extrinsic cell death signaling pathway in ECs during embryonic development has been substantially studied, the role of the intrinsic pathway has been less clearly defined. Due to the redundancy and large functional overlap of BCL2 family members, the developmental contribution of mitochondria-mediated cell death in the formation of the embryonic vascular system is difficult to elucidate. EC l-specific *Mcl1* knockout embryos present abnormal angiogenesis in the skin and die at E14.5 due to edema and hemorrhages [68]. Whereas mice lacking BAX or BAK only display mild developmental defects that result in lymphoid cell hyperplasia and increased platelet numbers in the adult [69, 70], more than 90% of *Bax*/*Bak* double knockout mice die perinatally due to multiple reasons, including improper formation of the aortic arches [29]. Interestingly, the cardiovascular system in the survivors seemed largely normal, even though they presented multiple defects related to insufficient apoptosis in other tissues [29, 71]. This result indicates that cell death via the intrinsic pathway is either not essential in embryonic ECs or can be compensated and executed by BAX/BAK-independent mechanisms.

Taken together, the above-mentioned studies so far suggest that EC death is dispensable for proper formation of the early embryonic cardiovascular system, but has to be actively inhibited to allow embryonic development. It might be that a lack of apoptosis can be compensated by other cell death modalities. Therefore, it remains to be elucidated, whether combined inhibition of multiple cell death pathways would also be tolerated in the developing vasculature.

## EC apoptosis during postnatal development

### Regression of the hyaloid vasculature is mediated by the BCL2 signaling pathway

The hyaloid vasculature and pupillary membrane are specialized vascular structures in the eye of the embryo that supply trophic support to the growing lens during embryogenesis. In parallel to the development of the postnatal retinal vascular system (see below), both the hyaloid vessels and pupillary membrane regress [72]. Persistent fetal vasculature (PFV), one of the most common developmental congenital ocular malformations, is a human disease that results from inefficient removal of the hyaloid vasculature [73]. Interestingly, hyaloid vessel, but not pupillary membrane regression, is inhibited in *Bax/Bax* double knockout mice, where these vessels are still present in adulthood [17]. These results indicate that apoptosis in ECs is required for hyaloid vessel regression during development.

Pupillary membrane, and likely hyaloid vessel regression as well, takes place in two phases. In the first phase, macrophages induce apoptosis in single ECs, for example via Wnt-7b signaling [74–76]. This is followed by subsequent lumen constriction and thus disturbances in blood flow that induce a second phase of EC apoptosis in larger vessel segments [77, 78]. *PU.1* mutant mice that lack resident macrophages [75] suffer from PFV , thus supporting a role of macrophages in the removal of these fetal vascular beds [9, 72, 74, 78]. Hyaloid vessel regression is also regulated by retinal neurons that express VEGFR2 and can titrate VEGF availability [8]. In this context, the BH3 only protein BIM is an essential inducer of apoptosis in ECs and normally suppressed by VEGF signaling [79, 80]. Therefore, hyaloid vessel regression is inhibited in *Bim*^*−/−*^ mice [80, 81]. In contrast, loss of the pro-apoptotic BH3 only protein BIK [82] or the antiapoptotic BCL2  [83] did not affect hyaloid vessel regression, indicating again redundancy of some of the BH3 family members.

### Apoptosis during angiogenesis in the mouse retina

The retina vasculature develops in utero in humans [73]. However, in mice, this vasculature develops postnatally during the first 3 weeks after birth [84]. Much of our knowledge of the different phases of angiogenesis (sprouting and growth, maturation and remodeling) has come from studying the neonatal mouse retina [85, 86], and different genetic mouse mutants have been analyzed to understand the contribution of cell death to these processes (Table 1). Starting at postnatal day (P) 1, vessel sprouts emerge from the optic nerve head at the center of the retina from where they expand radially towards the retina periphery until ~ P8 [85]. Whereas EC proliferation and vessel sprouting mainly take place at the leading edge of the growing vascular system, vessel maturation predominantly occurs more centrally. Nevertheless, this division is not absolute as EC proliferation is also observed in and around the central vein segments [87], and pruning vessels can be found throughout the vessel network [88]. A small subset of apoptotic ECs has been observed during retina angiogenesis [12, 88–90]. While these few apoptotic ECs are initially clustered around remodeling arteries, away from proliferative regions of the retina, they appear more evenly distributed over time as soon as EC proliferation ceases [12].

Maturing vessels become covered by mural cells that provide pro-survival factors such as ANG-1 [91] and VEGF [92]  to promote EC survival, vessel stability and integrity. In line, EC apoptosis during retina angiogenesis increased dramatically in the absence of pericytes [93]. In these conditions, the remaining vasculature presented defective blood–retinal barrier formation and vascular hemorrhages due to the upregulation of vascular destabilization factors in ECs, such as ANG-2, downstream of FOXO1 [93]. Consistently, EC apoptosis is decreased in pericyte-specific *Bim* knockout mice (where pericyte numbers are increased [94]), indicating that pericytes are crucial for EC survival during vessel growth. On the other hand, one study reported that pericytes promote EC apoptosis in the postnatal retina through the production of endosialin, whose expression is restricted to pericytes covering newly formed vessels [95]. Whether pericytes undergo cell death during physiological blood vessel pruning is also controversial. While certain studies indicate that pericytes stay attached to the empty basement membrane of pruning vessels [88, 90, 96], others report that pericytes undergo apoptosis [7] or migrate into adjacent vessel segments [97, 98]. Further studies will be required in the future to determine the role and adaptive fate of pericytes during vessel pruning.

A role for immune cells in EC apoptosis and clearance during vessel pruning has also been reported. Leukocytes and cytotoxic T cells are able to induce EC death during retinal vessel pruning via CD95L [99]. During regression of the hyaloid vasculature, or during blood vessel pruning in the trachea of postnatal pups (upon anti-VEGF treatment), macrophages are also seen in close proximity to remodeling vessels. In these cases, they contribute to the passive clearance of apoptotic ECs [98, 100]. However, both in the embryo hindbrain and postnatal retina, macrophages additionally contribute to lumen formation and vessel remodeling by promoting vessel anastomosis [101]. This suggests that they might have dual roles or that different macrophage populations have distinct functions. In the zebrafish brain vasculature, microglia have also been shown to associate with pruning vessels after ECs underwent apoptosis, thus also indicating that they do not actively contribute to EC death, but rather to their removal afterwards [102]. While those studies indicate a fundamental role of the immune compartment in proper blood vessel development, further research is required to determine the extent of active and passive contribution of the immune compartment to vessel remodeling in different vascular beds.

### Does EC apoptosis drive vessel pruning?

Whereas it is undebated that EC death accounts for the complete removal of fetal vascular systems, such as the hyaloid vasculature, different mechanisms might regulate partial vessel removal of a subset of vascular branches during postnatal development (vessel pruning). In addition to the studies mentioned above, where the potential contribution of pericytes and immune cell-driven induction of EC apoptosis and their contribution to vessel pruning were discussed [94, 95, 99, 101], one other hypothesis suggests that vessel pruning is primarily driven by EC migration and re-integration into neighboring vessel branches. We discuss below the contributions of both processes to postnatal vessel remodeling.

EC apoptosis has been implicated in certain conditions of vessel remodeling [90, 103–106] and in the regulation of capillary vessel diameter [12]. In the maturation phase of the retinal vasculature, characteristic avascular areas form around differentiating arteries. EC apoptosis is frequently observed in pruning vessels around arteries [12, 68, 88, 95] and several mouse mutants confirm a role of EC death in the regulation of vessel density. For example, mice lacking the pro-apoptotic BH3-only protein BIM [81] present increased vessel density in the retina. Loss of EC TIE1 [107] also leads to an increase in EC death and concomitant reduction in vessel density. A similar phenotype has been reported when regulators of the Wnt pathway, such as *Gpr177* (required for wnt secretion) and *Rspo3* (a wnt signaling enhancer) [90, 108] were knocked out in ECs. Interestingly, non-canonical Wnt signaling was further shown to regulate the response of ECs to shear stress, rather than cell death [109].

Even though EC death might affect vessel density, it is debated whether apoptosis under physiological conditions is required to initiate vessel pruning, as, despite a mild increase in the vascular area, vessel remodeling and maturation properly take place in the above-mentioned knockout mice and others. For example, even though EC apoptosis is increased in mice lacking the pro-survival protein MCL1, vessel regression is not affected [12]. In addition, vessel regression is unaffected in EC-specific *Casp8* knockout mice [13], even though EC apoptosis is reduced. Furthermore, *TRAIL*^−/−^ mice only show a mild and transient increase in vessel density that is recovered at P5, and a small decrease in the capillary free area surrounding arteries [110]. In the developing rat retina, no correlation between vessel removal and EC apoptosis could be found [89]. Consistently, while the majority of apoptotic cells in the developing mouse and zebrafish retinal vasculature are found in pruning vessels, only a small subset of them (around 5% to 15%) contained apoptotic ECs [12, 88, 102]. Up to date, analysis of retina angiogenesis in *Bak*^*−/−*^* Bax*^*EC/EC*^ mice (with full *Bak*, and EC-specific *Bax* deletion) has most directly evaluated the impact of EC death to vessel remodeling. Strikingly, *Bak*^*−/−*^* Bax*^*EC/EC*^ knockout  with full inhibition of apoptosis via the intrinsic cell death signaling pathway in ECs, delayed, but did not prevent vessel pruning around arteries [12]. In the capillary region, where apoptosis is more scattered, blocking apoptosis did not have any effect on vessel pruning either [12].

Elegant time-lapse imaging studies in zebrafish have ultimately revealed in vivo that EC apoptosis is not common during vessel pruning [105, 111, 112]. Instead, ECs migrate out of remodeling vessels and reintegrate into neighboring vessel segments, thus eliminating the need for EC removal by cell death. In this model, changes in blood flow and the resulting impact on shear–stress are the driving forces for vessel pruning [111]. EC migration in response to hemodynamic cues also occurs in the mouse retina [88]. Interestingly, the sensitivity of ECs to flow depends on non-canonical wnt signaling, as in its absence ECs show an increased sensitivity to flow which results in premature vessel pruning [109].

One possible explanation linking the cell death and migration hypothesis could be that ECs that detach from the extracellular matrix during migration undergo cell death due to anoikis, which might explain why the majority of apoptotic ECs are found in remodeling vessels. In this case, EC apoptosis might not be the primary cause of vessel pruning, but consequence of cellular detachment or secondary to impaired blood flow [105]. It should be noted that TUNEL or cleaved CASP-3 positive cells have a short half-life and the commonly observed shedding of apoptotic ECs into the vascular lumen and their quick clearance by macrophages further complicates the precise evaluation of the kinetics of the above-mentioned processes. In addition, as our current knowledge of vessel remodeling is based on studies from both wildtype and mutant mice, caution should be taken by comparing physiologically and artificially induced vessel remodeling. Taken together, successful vessel pruning is likely a multi-step process involving EC migration, reintegration into neighboring vessels and a minor contribution of EC apoptosis.

## Cell death-independent functions of the apoptosis signaling machinery in ECs

Accumulating evidences suggest that molecules of the cell death signaling machinery in ECs (and other cell types) can act beyond their classic apoptotic function. Deletion of TAK1 in ECs does not just lead to increased TNF-dependent EC apoptosis, but also to TNF-independent defects in EC migration [55]. Furthermore, the loss of the IAP survivin in ECs does not just lead to increased EC death in the developing embryo, but also to defects in neural tube closure. Those are not solely attributable to tissue hypoxia resulting from vascular malformations, but to cell death-independent changes in EC derived growth factor secretion that otherwise supports neural tube closure under physiological conditions [113]. Similarly, ECs of *Casp8* knockout embryos display an enhanced cytokine expression profile, which is independent of RIPK3 and necroptosis [114]. These studies suggest that certain cell death signaling molecules also regulate additional, non-cell death-related EC properties and functions such as growth factor and cytokine expression, which might have fundamental roles during tissue development. They also raise the question of whether embryonic lethality in diverse knockout mice related to the cell death signaling pathway is solely due to dysregulated apoptosis/necroptosis or further supported by other, non-cell death related vascular dysfunctions.

During postnatal angiogenesis, several studies have shown that the pro-cell death signaling machinery does not promote apoptosis, but regulates vessel development independent of cell death. In contrast to many other cell types, the pro-apoptotic BH3-only protein PUMA, though strongly expressed in ECs [15], does not induce apoptosis [115]. Instead, it regulates EC proliferation, autophagy and blood vessel development in the retina via ERK signaling [15]. CD95 is also expressed in brain and retina ECs during postnatal development, and angiogenesis is delayed in EC-specific *CD95* knockout mice [14]. In ECs, co-immunoprecipitation experiments revealed that CD95 interacts with SFK and p85. In line, CD95L stimulation of HUVECs leads to the phosphorylation and activation of Akt and ERK, and inhibition of Akt blocks CD95L induced EC proliferation [14]. In addition to its physiological role during angiogenesis in the central nervous system, activation of CD95 with the agonistic anti-CD95 mAb Jo2 was able to induce immune cell infiltration and angiogenesis in an in vivo Matrigel assay [116], indicating that CD95 signaling might also regulate inflammation induced angiogenesis. Recently, CASP-8 has also been shown to regulate angiogenesis in the retina in a cell death-independent way. Even though lethal during embryonic development, knockout of *Casp8* in ECs during postnatal development did not affect mouse survival, thus indicating that after a critical time window of vasculogenesis and angiogenesis during embryonic development, postnatal ECs are more resistant to cell death in the absence of CASP-8. In fact, postnatal deletion of *Casp8* in ECs only led to a mild delay in angiogenesis, which was dependent on RIPK3, but not MLKL and therefore necroptosis [13]. In line, RIPK1 and RIPK3 have been shown to positively regulate the response of ECs to VEGF stimulation in the bead sprouting assay, as inhibition of either of them ameliorated the VEGF induced sprouting response [117]. In both of the studies indicated above, this effect was mediated by increased p38 signaling, a known inhibitor of angiogenesis [118].

Even though the above-mentioned findings support a cell death-independent role of the cell death signaling machinery, the effect of death ligand stimulation of ECs per se in an in vitro setting is controversial and largely context dependent. Whereas some studies report that stimulation with TRAIL induces apoptosis in HUVECs [119] and has anti-angiogenic properties [120], other studies find that TRAIL stimulates angiogenesis [121]. In addition, and even in the absence of CASP-8, TNF-α and TRAIL (potent inducers of necroptosis in the absence of CASP-8 in other cell types [42, 122–124]) did not induce cell death in ECs in vitro [13]. Similar findings have been reported for CD95L. Even though CD95 is expressed on the surface of ECs, they are resistant to CD95L-induced cell death in vitro [125–127]. In HUVECs, this is mediated by association of monomeric CD95 with cMet and a strong expression and recruitment of c-FLIP to the DISC, therefore preventing CD95 oligomerization and DISC activation [128]. Interestingly, dimerized CD95 is detected when ECs lose matrix attachment and undergo anoikis [128] or when Akt signaling is inhibited [129], which highlights again that apoptosis might be a consequence, not cause, of vascular removal. To further explain why ECs seem surprisingly resistant to cell death, one study suggests that HUVECs treated with LPS secrete cleaved CASP-3 fragments into the supernatant, thus actively escaping from apoptosis [130]. Additional non-apoptotic, but barrier-promoting functions of CASP-3 have been reported in lung microvascular ECs [131]. Even though apoptosis was reported to be increased in the retina vasculature of *Bcl2*^−/−^ mice in vivo [83], isolated retina ECs from these mice did not show increased cell death under basal conditions but defects in cell adhesion, migration and capillary morphogenesis [132]. Thus, this data suggests that *Bcl2*^−/−^ ECs behave differently in processes of angiogenesis and that an environmental factor not present in culture would be required to induce apoptosis in vivo.

Taken together, these studies highlight the exceptional cell death-independent functions of the apoptosis machinery in ECs during physiological blood vessel formation. They further strengthen the hypothesis of a timely appropriate requirement of pro-survival factors, such as CASP-8, in the developing endothelium during the course of vessel formation.

## EC death in pathology: a focus on oxygen induced retinopathy

ROP  and proliferative diabetic retinopathy (PDR) are diseases characterized by pathological EC apoptosis and vessel obliteration that is followed by an equally pathological boost of neo-vascularization which often results in the formation of disturbed and leaky vascular tufts [133, 134]. Vessels growing into the vitreous often lead to vision impairment, hemorrhage, scarring and in the worst case, retinal detachment [135]. ROP can be recapitulated in the oxygen-induced retinopathy (OIR) model in mice or rats [133, 134]. In this model, newborn mice are briefly exposed to hyperoxic conditions, which will lead to the regression of all capillaries in the central retina. Upon return to normal oxygen conditions, these areas become hypoxic, leading to increased production of proangiogenic factors, such as VEGF, PlGF and IGF-1 and thus pathological neoangiogenesis [136–138]. EC apoptosis accounts for the initial hyperoxia-induced vaso-obliteration, thus rendering the OIR model a useful system for the study of pathological EC death and regression [133, 139]. In addition, the malformed neo-vasculature undergoes spontaneous regression over time [85] which will eventually lead to the resolution of the vascular malformations. Similar to ROP, PDR is characterized by retinal–capillary non-perfusion that results in EC and pericyte apoptosis [140]. Again, this capillary loss results in the formation of focal ischemic-areas that induce an increased production of cytokines and pro-angiogenic factors such as VEGF-A , therefore resulting in increased pathological angiogenesis [141].

Several studies indicate that vaso-obliteration and EC apoptosis in the OIR model are regulated by the intrinsic cell death signaling pathway. *Bim*^−/−^ mice are resistant to hyperoxia induced vessel regression [81]. On the other hand, vaso-obliteration did not depend on BCL1, even though *Bcl2*^*−/−*^ mice presented a reduced amount of pathological neovascular tufts [83]. Strikingly, however, this phenotype was not recapitulated in EC specific *Bcl-2*^*−/−*^ mice, where vaso-obliteration was increased, but neovascularization unchanged compared to control littermates [142], indicating that BCL2 in other cell types also modulated OIR. More recently, it has been shown that revascularization in *Bak*^*−/−*^*Bax*^*EC/EC*^ mice during OIR is increased. Even though ECs in these mice are protected from apoptosis, vessel obliteration and ischemia still occured. However, ECs within closed and unperfused vessels that were protected from apoptosis were able to rebuild a functional vascular system in response to pro-angiogenic stimulation faster compared to control mice, thus resulting in increased revascularization upon return to normoxia [143].

In contrast to its mild contribution to vessel pruning during physiological angiogenesis, the extrinsic cell death signaling pathway regulates EC death and vessel resolution in the OIR model. In the rat, treatment with a CD95L-neutralizing antibody reduced vaso-obliteration in the OIR model [99]. However, *CD95L* loss of function mice (*Fasl*^*gld/gld*^) show normal vaso-obliteration in the OIR model, but the formation of neovascular tufts was increased [144, 145]. This was accompanied by having fewer TUNEL + cells in the tuft areas, indicating that CD95 signaling might limit pathologic neovascularization by inducing EC apoptosis. *TRAIL*^*−/−*^ mice did not show any impairment in vaso-obliteration, however, similar to *Fasl*^*gld/gld*^ mutant mice, neovascularization was increased [110]. Furthermore, tuft regression was delayed, indicating that TRAIL was required to limit both excessive neovascularization and to promote resolution of malformed vessels. Consistent with its role as a pro-survival factor, vaso-obliteration in the OIR model was increased in EC specific *Casp8* knockout mice [13]. Of benefit, as CASP-8 regulates angiogenesis in a cell death-independent way, tuft formation was also decreased in the OIR model [13], suggesting that regulating CASP-8 in ECs could be beneficial for inducing or preventing vessel growth.

Taken together, our knowledge based on studies of the OIR model indicates that EC apoptosis can be a major contributor to neovascular diseases such as ROP and PDR. It should be noted that ROP is unique and different from other pathological neovascular conditions, as the vessels in the developing retina are still immature and thus dependent on VEGF signaling. In line, adult mice are resistant to OIR. This is consistent with, for example, the timely role of BCL2 and CASP-8 in early development, as discussed before. Hence, it also supports the finding that ECs possess a differential sensitivity and phenotypic outcome towards cell death by different signaling pathways. The above-mentioned studies, together with the fact that the extrinsic cell death signaling pathway also regulates cytokine production and thus potentially inflammation driven neo-angiogenesis, highlight the complex contribution of death receptor regulated signaling in ECs during pathological angiogenesis—a novel field that requires further investigation.

## Targeting the cell death signaling machinery in ECS during tumor angiogenesis

Growing tumors become vascularized with blood vessels to promote tumor oxygenation, nutrient delivery and ultimately metastatic spread [146]. Anti-angiogenic tumor therapy initially aimed to ‘starve tumors to death’ by inducing massive vessel regression [147]. Similar to physiological vessel development, TIE-1, EVI (the protein encoded by *Gpr177*) and R-spondin-3 have been shown to promote tumor angiogenesis. Therefore, deletion of these pro-survival factors in ECs has been shown to reduce tumor angiogenesis in B16-xenograft and lewis lung carcinoma (LLC) models [90, 107, 108]. In addition, as a subset of tumor vessels in expanding tumors remains immature, VEGF withdrawal strategies might be exploited, similar to treatment of ROP, for enforcing vessel regression [148]. However, as already indicated, these treatments only target the most immature vessels, while leaving behind a more mature and stable vascular network [149]. Therefore, new strategies are required to promote full regression of the tumor vasculature.

Whereas activation of the cell death signaling machinery in tumor cells has been in the center of anti-cancer therapy in the last decades [150, 151], studies targeting cell death signaling pathways in ECs to force vessel regression are just recently starting to emerge. Hereby, targeting TAK1 (which is known to inhibit apoptosis) in ECs seems to be a promising strategy. Specific deletion of TAK1 in ECs significantly reduced tumor growth in an EL4 lymphoma and LLC model that form tumors that are refractory to anti-VEGF therapy [18, 152]. Deletion of TAK1 after the tumor had already formed also lead to a significant regression of the tumor tissue due to increased EC apoptosis. However, vascular leakage, hemorrhage formation, and tumor hypoxia were increased [18]. Another study reported that TAK1 deletion induced EC necroptosis and, therefore, increased metastatic spread, as tumor cell extravasation was promoted in the leaky vasculature [153]. Consistently, several other studies have reported that EC necroptosis, or activation of the necroptosis signaling machinery in ECs, rather favor metastatic spread. For example, tumor cells extravasate by inducing necroptosis of ECs via DR6, which is further increased in EC specific *Casp8* knockout mice, where induction of necroptosis is facilitated [154]. In contrast, inhibition of RIPK1 and RIPK3 reduces metastasis formation and vascular leakage, even though contradictory data exists on whether this is via inhibition of necroptosis, or inhibition of cell death-independent functions of RIP-kinases [117, 154].

Taken together, EC death in the tumor vasculature clearly has its advantages and disadvantages and further studies are required to decipher the potential of induced EC death in cancer therapy. Hereby, it is likely that its effects might be different in the primary tumor and sites of tumor metastasis and also depend on the precise cell death modality, for example EC apoptosis vs. necroptosis.

## Conclusions and future perspective

**Fig. 2:**
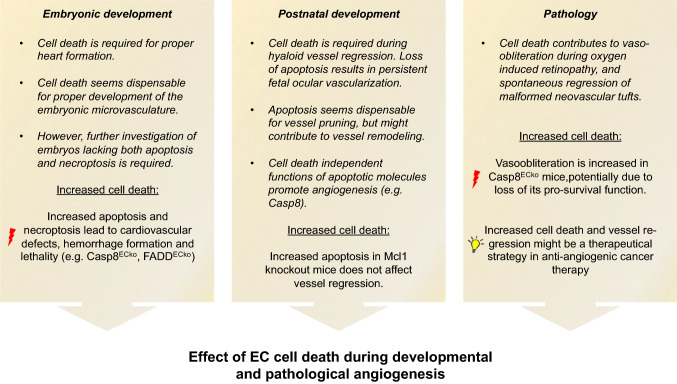
Contribution and function of cell death signaling molecules in ECs during vessel development and pathology. Apoptosis-related molecules can act in a cell death-dependent and -independent manner in ECs. Red lightnings symbols indicate negative effects, and light bulbs symbol indicates positive effects, of increased cell death signaling in ECs.

A collection of numerous studies over the last 20 years has shaped our understanding of EC death during blood vessel development and also significantly contributed to our knowledge about pathological EC death to diseases such as ROP. Taken together, those studies highlight the differential function of cell death signaling molecules in ECs during these processes (Fig. 2). Yet, it is important to note that most of our interpretations are based on studies in the mouse retina. Therefore, further research is required to understand the differential contribution of EC death in different vascular beds and different developmental stages: How is it possible, that the same pro-survival factors, such as CASP-8, are essential during vessel formation in the embryo, but dispensable at later developmental stages? And what is controlling the switch from a deathly cell death signaling machinery to the proangiogenic functions of the same molecules?

In this context, the role of vessel-associated cells, such as pericytes and macrophages that could substantially alter the sensitivity of ECs to cell death requires further attention. In addition, regulation of differential cell behavior by the physical properties of the environment, for example by the extracellular matrix (ECM) is gaining increasing attention. To answer these questions, improved invivo systems, combined with mathematical modeling, are required to study the interaction of ECs within the vessel sprout and its surrounding tissue during angiogenesis to precisely define the timely manner of EC migration, apoptosis, and vessel pruning.

Last but not least, further work is required to translate our knowledge of EC death under physiological conditions to pathological vessel regression. Do they follow the same mechanistic principles? And is the cell death signaling machinery required to maintain physiological vessel homeostasis in the quiescent, adult vasculature? Answering these questions  will contribute to the fundamental understanding of the mechanisms regulating vessel formation and will contribute to develop and improve the efficacy of anti-angiogenic therapies.

## Abbreviations

EC: Endothelial cell
OIR: Oxygen-induced retinopathy

## References

[1] Potente M, Gerhardt H, Carmeliet P (2011). Basic and therapeutic aspects of angiogenesis. Cell.

[2] Modlich U, Kaup FJ, Augustin HG (1996). Cyclic angiogenesis and blood vessel regression in the ovary: blood vessel regression during luteolysis involves endothelial cell detachment and vessel occlusion. Lab Invest.

[3] Ding YH, Li J, Zhou Y, Rafols JA, Clark JC, Ding Y (2006). Cerebral angiogenesis and expression of angiogenic factors in aging rats after exercise. Curr Neurovasc Res.

[4] Morland C, Andersson KA, Haugen OP, Hadzic A, Kleppa L, Gille A, Rinholm JE, Palibrk V, Diget EH, Kennedy LH, Stolen T, Hennestad E, Moldestad O, Cai Y, Puchades M, Offermanns S, Vervaeke K, Bjoras M, Wisloff U, Storm-Mathisen J, Bergersen LH (2017). Exercise induces cerebral VEGF and angiogenesis via the lactate receptor HCAR1. Nat Commun.

[5] Tonnesen MG, Feng X, Clark RA (2000). Angiogenesis in wound healing. J Investig Dermatol Symp Proc.

[6] Weis SM, Cheresh DA (2011). Tumor angiogenesis: molecular pathways and therapeutic targets. Nat Med.

[7] Taniguchi H, Kitaoka T, Gong H, Amemiya T (1999). Apoptosis of the hyaloid artery in the rat eye. Ann Anat.

[8] Yoshikawa Y, Yamada T, Tai-Nagara I, Okabe K, Kitagawa Y, Ema M, Kubota Y (2016). Developmental regression of hyaloid vasculature is triggered by neurons. J Exp Med.

[9] Ito M, Yoshioka M (1999). Regression of the hyaloid vessels and pupillary membrane of the mouse. Anat Embryol (Berl).

[10] Hiruma T, Hirakow R (1995). Formation of the pharyngeal arch arteries in the chick-embryo—observations of corrosion casts by scanning electron-microscopy. Anat Embryol.

[11] Rana MS, Sizarov A, Christoffels VM, Moorman AFM (2014). Development of the human aortic arch system captured in an interactive three-dimensional reference model. Am J Med Genet A.

[12] Watson EC, Koenig MN, Grant ZL, Whitehead L, Trounson E, Dewson G, Coultas L (2016). Apoptosis regulates endothelial cell number and capillary vessel diameter but not vessel regression during retinal angiogenesis. Development.

[13] Tisch N, Freire-Valls A, Yerbes R, Paredes I, La Porta S, Wang X, Martin-Perez R, Castro L, Wong WW, Coultas L, Strilic B, Grone HJ, Hielscher T, Mogler C, Adams RH, Heiduschka P, Claesson-Welsh L, Mazzone M, Lopez-Rivas A, Schmidt T, Augustin HG, Ruiz de Almodovar C (2019). Caspase-8 modulates physiological and pathological angiogenesis during retina development. J Clin Invest.

[14] Chen S, Tisch N, Kegel M, Yerbes R, Hermann R, Hudalla H, Zuliani C, Gulculer GS, Zwadlo K, von Engelhardt J, Ruiz de Almodovar C, Martin-Villalba A (2017). CNS Macrophages control neurovascular development via CD95L. Cell reports.

[15] Zhang F, Li Y, Tang Z, Kumar A, Lee C, Zhang L, Zhu C, Klotzsche-von Ameln A, Wang B, Gao Z, Zhang S, Langer HF, Hou X, Jensen L, Ma W, Wong W, Chavakis T, Liu Y, Cao Y, Li X (2012). Proliferative and survival effects of PUMA promote angiogenesis. Cell reports.

[16] Zhang C, Asnaghi L, Gongora C, Patek B, Hose S, Ma B, Fard MA, Brako L, Singh K, Goldberg MF, Handa JT, Lo WK, Eberhart CG, Zigler JS, Sinha D (2011). A developmental defect in astrocytes inhibits programmed regression of the hyaloid vasculature in the mammalian eye. Eur J Cell Biol.

[17] Hahn P, Lindsten T, Tolentino M, Thompson CB, Bennett J, Dunaief JL (2005). Persistent fetal ocular vasculature in mice deficient in bax and bak. Arch Ophthalmol.

[18] Naito H, Iba T, Wakabayashi T, Tai-Nagara I, Suehiro JI, Jia W, Eino D, Sakimoto S, Muramatsu F, Kidoya H, Sakurai H, Satoh T, Akira S, Kubota Y, Takakura N (2019). TAK1 Prevents endothelial apoptosis and maintains vascular integrity. Dev Cell.

[19] Green DR, Llambi F (2015). Cell death signaling. Cold Spring Harb Perspect Biol.

[20] Warren CFA, Wong-Brown MW, Bowden NA (2019). BCL-2 family isoforms in apoptosis and cancer. Cell Death Dis.

[21] Vaux DL, Cory S, Adams JM (1988). Bcl-2 gene promotes haemopoietic cell survival and cooperates with c-myc to immortalize pre-B cells. Nature.

[22] Huang DC, Strasser A (2000). BH3-Only proteins-essential initiators of apoptotic cell death. Cell.

[23] Pena-Blanco A, Garcia-Saez AJ (2018). Bax, Bak and beyond—mitochondrial performance in apoptosis. FEBS J.

[24] Jurgensmeier JM, Xie Z, Deveraux Q, Ellerby L, Bredesen D, Reed JC (1998). Bax directly induces release of cytochrome c from isolated mitochondria. Proc Natl Acad Sci USA.

[25] Kluck RM, Bossy-Wetzel E, Green DR, Newmeyer DD (1997). The release of cytochrome c from mitochondria: a primary site for Bcl-2 regulation of apoptosis. Science.

[26] Letai A, Bassik MC, Walensky LD, Sorcinelli MD, Weiler S, Korsmeyer SJ (2002). Distinct BH3 domains either sensitize or activate mitochondrial apoptosis, serving as prototype cancer therapeutics. Cancer Cell.

[27] Llambi F, Moldoveanu T, Tait SW, Bouchier-Hayes L, Temirov J, McCormick LL, Dillon CP, Green DR (2011). A unified model of mammalian BCL-2 protein family interactions at the mitochondria. Mol Cell.

[28] Llambi F, Wang YM, Victor B, Yang M, Schneider DM, Gingras S, Parsons MJ, Zheng JH, Brown SA, Pelletier S, Moldoveanu T, Chen T, Green DR (2016). BOK Is a non-canonical BCL-2 family effector of apoptosis regulated by ER-associated degradation. Cell.

[29] Ke FFS, Vanyai HK, Cowan AD, Delbridge ARD, Whitehead L, Grabow S, Czabotar PE, Voss AK, Strasser A (2018). Embryogenesis and adult life in the absence of intrinsic apoptosis effectors BAX, BAK, and BOK. Cell.

[30] Li P, Nijhawan D, Budihardjo I, Srinivasula SM, Ahmad M, Alnemri ES, Wang X (1997). Cytochrome c and dATP-dependent formation of Apaf-1/caspase-9 complex initiates an apoptotic protease cascade. Cell.

[31] Neamati N, Fernandez A, Wright S, Kiefer J, McConkey DJ (1995). Degradation of lamin B1 precedes oligonucleosomal DNA fragmentation in apoptotic thymocytes and isolated thymocyte nuclei. J Immunology.

[32] Kayalar C, Ord T, Testa MP, Zhong LT, Bredesen DE (1996). Cleavage of actin by interleukin 1 beta-converting enzyme to reverse DNase I inhibition. Proc Natl Acad Sci USA.

[33] Kaufmann SH, Desnoyers S, Ottaviano Y, Davidson NE, Poirier GG (1993). Specific proteolytic cleavage of poly(ADP-ribose) polymerase: an early marker of chemotherapy-induced apoptosis. Can Res.

[34] Lips J, Kaina B (2001). DNA double-strand breaks trigger apoptosis in p53-deficient fibroblasts. Carcinogenesis.

[35] Sansome C, Zaika A, Marchenko ND, Moll UM (2001). Hypoxia death stimulus induces translocation of p53 protein to mitochondria. Detection by immunofluorescence on whole cells. FEBS Lett.

[36] Wensveen FM, Alves NL, Derks IA, Reedquist KA, Eldering E (2011). Apoptosis induced by overall metabolic stress converges on the Bcl-2 family proteins Noxa and Mcl-1. Apoptosis: Int J Program Cell Death.

[37] Lavrik I, Golks A, Krammer PH (2005). Death receptor signaling. J Cell Sci.

[38] Siegmund D, Lang I, Wajant H (2017). Cell death-independent activities of the death receptors CD95, TRAILR1, and TRAILR2. FEBS J.

[39] Slee EA, Keogh SA, Martin SJ (2000). Cleavage of BID during cytotoxic drug and UV radiation-induced apoptosis occurs downstream of the point of Bcl-2 action and is catalysed by caspase-3: a potential feedback loop for amplification of apoptosis-associated mitochondrial cytochrome c release. Cell Death Differ.

[40] Kataoka T, Schroter M, Hahne M, Schneider P, Irmler M, Thome M, Froelich CJ, Tschopp J (1998). FLIP prevents apoptosis induced by death receptors but not by perforin/granzyme B, chemotherapeutic drugs, and gamma irradiation. J Immunology.

[41] Oberst A, Dillon CP, Weinlich R, McCormick LL, Fitzgerald P, Pop C, Hakem R, Salvesen GS, Green DR (2011). Catalytic activity of the caspase-8-FLIP(L) complex inhibits RIPK3-dependent necrosis. Nature.

[42] Holler N, Zaru R, Micheau O, Thome M, Attinger A, Valitutti S, Bodmer JL, Schneider P, Seed B, Tschopp J (2000). Fas triggers an alternative, caspase-8-independent cell death pathway using the kinase RIP as effector molecule. Nat Immunol.

[43] Cho YS, Challa S, Moquin D, Genga R, Ray TD, Guildford M, Chan FK (2009). Phosphorylation-driven assembly of the RIP1-RIP3 complex regulates programmed necrosis and virus-induced inflammation. Cell.

[44] Feng S, Yang Y, Mei Y, Ma L, Zhu DE, Hoti N, Castanares M, Wu M (2007). Cleavage of RIP3 inactivates its caspase-independent apoptosis pathway by removal of kinase domain. Cell Signal.

[45] Lin Y, Devin A, Rodriguez Y, Liu ZG (1999). Cleavage of the death domain kinase RIP by caspase-8 prompts TNF-induced apoptosis. Genes Dev.

[46] O’Donnell MA, Perez-Jimenez E, Oberst A, Ng A, Massoumi R, Xavier R, Green DR, Ting AT (2011). Caspase 8 inhibits programmed necrosis by processing CYLD. Nat Cell Biol.

[47] Sun L, Wang H, Wang Z, He S, Chen S, Liao D, Wang L, Yan J, Liu W, Lei X, Wang X (2012). Mixed lineage kinase domain-like protein mediates necrosis signaling downstream of RIP3 kinase. Cell.

[48] Zhao J, Jitkaew S, Cai ZY, Choksi S, Li QN, Luo J, Liu ZG (2012). Mixed lineage kinase domain-like is a key receptor interacting protein 3 downstream component of TNF-induced necrosis. Proc Natl Acad Sci USA.

[49] Dondelinger Y, Declercq W, Montessuit S, Roelandt R, Goncalves A, Bruggeman I, Hulpiau P, Weber K, Sehon CA, Marquis RW, Bertin J, Gough PJ, Savvides S, Martinou JC, Bertrand MJ, Vandenabeele P (2014). MLKL compromises plasma membrane integrity by binding to phosphatidylinositol phosphates. Cell Rep.

[50] Ros U, Pena-Blanco A, Hanggi K, Kunzendorf U, Krautwald S, Wong WW, Garcia-Saez AJ (2017). Necroptosis execution is mediated by plasma membrane nanopores independent of calcium. Cell reports.

[51] Buckingham M, Meilhac S, Zaffran S (2005). Building the mammalian heart from two sources of myocardial cells. Nat Rev Genet.

[52] Schmidt A, Brixius K, Bloch W (2007). Endothelial precursor cell migration during vasculogenesis. Circ Res.

[53] Fisher SA, Langille BL, Srivastava D (2000). Apoptosis during cardiovascular development. Circ Res.

[54] Blonska M, Shambharkar PB, Kobayashi M, Zhang D, Sakurai H, Su B, Lin X (2005). TAK1 is recruited to the tumor necrosis factor-alpha (TNF-alpha) receptor 1 complex in a receptor-interacting protein (RIP)-dependent manner and cooperates with MEKK3 leading to NF-kappaB activation. J Biol Chem.

[55] Morioka S, Inagaki M, Komatsu Y, Mishina Y, Matsumoto K, Ninomiya-Tsuji J (2012). TAK1 kinase signaling regulates embryonic angiogenesis by modulating endothelial cell survival and migration. Blood.

[56] Yeh WC, Itie A, Elia AJ, Ng M, Shu HB, Wakeham A, Mirtsos C, Suzuki N, Bonnard M, Goeddel DV, Mak TW (2000). Requirement for Casper (c-FLIP) in regulation of death receptor-induced apoptosis and embryonic development. Immunity.

[57] Varfolomeev EE, Schuchmann M, Luria V, Chiannilkulchai N, Beckmann JS, Mett IL, Rebrikov D, Brodianski VM, Kemper OC, Kollet O, Lapidot T, Soffer D, Sobe T, Avraham KB, Goncharov T, Holtmann H, Lonai P, Wallach D (1998). Targeted disruption of the mouse Caspase 8 gene ablates cell death induction by the TNF receptors, Fas/Apo1, and DR3 and is lethal prenatally. Immunity.

[58] Yeh WC, de la Pompa JL, McCurrach ME, Shu HB, Elia AJ, Shahinian A, Ng M, Wakeham A, Khoo W, Mitchell K, El-Deiry WS, Lowe SW, Goeddel DV, Mak TW (1998). FADD: essential for embryo development and signaling from some, but not all, inducers of apoptosis. Science.

[59] Dillon CP, Oberst A, Weinlich R, Janke LJ, Kang TB, Ben-Moshe T, Mak TW, Wallach D, Green DR (2012). Survival function of the FADD-CASPASE-8-cFLIP(L) complex. Cell reports.

[60] Kaiser WJ, Upton JW, Long AB, Livingston-Rosanoff D, Daley-Bauer LP, Hakem R, Caspary T, Mocarski ES (2011). RIP3 mediates the embryonic lethality of caspase-8-deficient mice. Nature.

[61] Alvarez-Diaz S, Dillon CP, Lalaoui N, Tanzer MC, Rodriguez DA, Lin A, Lebois M, Hakem R, Josefsson EC, O'Reilly LA, Silke J, Alexander WS, Green DR, Strasser A (2016). The pseudokinase MLKL and the kinase RIPK3 have distinct roles in autoimmune disease caused by loss of death-receptor-induced apoptosis. Immunity.

[62] Kang TB, Ben-Moshe T, Varfolomeev EE, Pewzner-Jung Y, Yogev N, Jurewicz A, Waisman A, Brenner O, Haffner R, Gustafsson E, Ramakrishnan P, Lapidot T, Wallach D (2004). Caspase-8 serves both apoptotic and nonapoptotic roles. Journal of immunology.

[63] Fan C, Pu W, Wu X, Zhang X, He L, Zhou B, Zhang H (2016). Lack of FADD in Tie-2 expressing cells causes RIPK3-mediated embryonic lethality. Cell Death Dis.

[64] Peltzer N, Rieser E, Taraborrelli L, Draber P, Darding M, Pernaute B, Shimizu Y, Sarr A, Draberova H, Montinaro A, Martinez-Barbera JP, Silke J, Rodriguez TA, Walczak H (2014). HOIP deficiency causes embryonic lethality by aberrant TNFR1-mediated endothelial cell death. Cell reports.

[65] Adachi M, Suematsu S, Kondo T, Ogasawara J, Tanaka T, Yoshida N, Nagata S (1995). Targeted mutation in the Fas gene causes hyperplasia in peripheral lymphoid organs and liver. Nat Genet.

[66] Diehl GE, Yue HH, Hsieh K, Kuang AA, Ho M, Morici LA, Lenz LL, Cado D, Riley LW, Winoto A (2004). TRAIL-R as a negative regulator of innate immune cell responses. Immunity.

[67] Rothe J, Mackay F, Bluethmann H, Zinkernagel R, Lesslauer W (1994). Phenotypic analysis of TNFR1-deficient mice and characterization of TNFR1-deficient fibroblasts in vitro. Circ Shock.

[68] Watson EC, Whitehead L, Adams RH, Dewson G, Coultas L (2016). Endothelial cell survival during angiogenesis requires the pro-survival protein MCL1. Cell Death Differ.

[69] Mason KD, Carpinelli MR, Fletcher JI, Collinge JE, Hilton AA, Ellis S, Kelly PN, Ekert PG, Metcalf D, Roberts AW, Huang DC, Kile BT (2007). Programmed anuclear cell death delimits platelet life span. Cell.

[70] Knudson CM, Tung KS, Tourtellotte WG, Brown GA, Korsmeyer SJ (1995). Bax-deficient mice with lymphoid hyperplasia and male germ cell death. Science.

[71] Lindsten T, Ross AJ, King A, Zong WX, Rathmell JC, Shiels HA, Ulrich E, Waymire KG, Mahar P, Frauwirth K, Chen Y, Wei M, Eng VM, Adelman DM, Simon MC, Ma A, Golden JA, Evan G, Korsmeyer SJ, MacGregor GR, Thompson CB (2000). The combined functions of proapoptotic Bcl-2 family members bak and bax are essential for normal development of multiple tissues. Mol Cell.

[72] Mitchell CA, Risau W, Drexler HC (1998). Regression of vessels in the tunica vasculosa lentis is initiated by coordinated endothelial apoptosis: a role for vascular endothelial growth factor as a survival factor for endothelium. Dev Dyn.

[73] Goldberg MF (1997). Persistent fetal vasculature (PFV): an integrated interpretation of signs and symptoms associated with persistent hyperplastic primary vitreous (PHPV). LIV Edward Jackson Memorial Lecture. Am J Ophthalmol.

[74] Lang RA, Bishop JM (1993). Macrophages are required for cell death and tissue remodeling in the developing mouse eye. Cell.

[75] Lobov IB, Rao S, Carroll TJ, Vallance JE, Ito M, Ondr JK, Kurup S, Glass DA, Patel MS, Shu W, Morrisey EE, McMahon AP, Karsenty G, Lang RA (2005). WNT7b mediates macrophage-induced programmed cell death in patterning of the vasculature. Nature.

[76] Rao S, Lobov IB, Vallance JE, Tsujikawa K, Shiojima I, Akunuru S, Walsh K, Benjamin LE, Lang RA (2007). Obligatory participation of macrophages in an angiopoietin 2-mediated cell death switch. Development.

[77] Meeson A, Palmer M, Calfon M, Lang R (1996). A relationship between apoptosis and flow during programmed capillary regression is revealed by vital analysis. Development.

[78] Meeson AP, Argilla M, Ko K, Witte L, Lang RA (1999). VEGF deprivation-induced apoptosis is a component of programmed capillary regression. Development.

[79] Naik E, O'Reilly LA, Asselin-Labat ML, Merino D, Lin A, Cook M, Coultas L, Bouillet P, Adams JM, Strasser A (2011). Destruction of tumor vasculature and abated tumor growth upon VEGF blockade is driven by proapoptotic protein Bim in endothelial cells. J Exp Med.

[80] Koenig MN, Naik E, Rohrbeck L, Herold MJ, Trounson E, Bouillet P, Thomas T, Voss AK, Strasser A, Coultas L (2014). Pro-apoptotic BIM is an essential initiator of physiological endothelial cell death independent of regulation by FOXO3. Cell Death Differ.

[81] Wang S, Park S, Fei P, Sorenson CM (2011). Bim is responsible for the inherent sensitivity of the developing retinal vasculature to hyperoxia. Dev Biol.

[82] Coultas L, Bouillet P, Stanley EG, Brodnicki TC, Adams JM, Strasser A (2004). Proapoptotic BH3-only Bcl-2 family member Bik/Blk/Nbk is expressed in hemopoietic and endothelial cells but is redundant for their programmed death. Mol Cell Biol.

[83] Wang S, Sorenson CM, Sheibani N (2005). Attenuation of retinal vascular development and neovascularization during oxygen-induced ischemic retinopathy in Bcl-2-/- mice. Dev Biol.

[84] Fruttiger M (2002). Development of the mouse retinal vasculature: angiogenesis versus vasculogenesis. Invest Ophthalmol Vis Sci.

[85] Stahl A, Connor KM, Sapieha P, Chen J, Dennison RJ, Krah NM, Seaward MR, Willett KL, Aderman CM, Guerin KI, Hua J, Lofqvist C, Hellstrom A, Smith LEH (2010). The mouse retina as an angiogenesis model. Invest Ophth Vis Sci.

[86] Uemura AK, Kusuhara S, Katsuta H, Nishikawa S (2006). Angiogenesis in the mouse retina: A model system for experimental manipulation. Exp Cell Res.

[87] Ehling M, Adams S, Benedito R, Adams RH (2013). Notch controls retinal blood vessel maturation and quiescence. Development.

[88] Franco CA, Jones ML, Bernabeu MO, Geudens I, Mathivet T, Rosa A, Lopes FM, Lima AP, Ragab A, Collins RT, Phng LK, Coveney PV, Gerhardt H (2015). Dynamic endothelial cell rearrangements drive developmental vessel regression. Plos Biol.

[89] Hughes S, Chan-Ling TL (2000). Roles of endothelial cell migration and apoptosis in vascular remodeling during development of the central nervous system. Microcirculation.

[90] Korn C, Scholz B, Hu J, Srivastava K, Wojtarowicz J, Arnsperger T, Adams RH, Boutros M, Augustin HG, Augustin I (2014). Endothelial cell-derived non-canonical Wnt ligands control vascular pruning in angiogenesis. Development.

[91] Augustin HG, Koh GY, Thurston G, Alitalo K (2009). Control of vascular morphogenesis and homeostasis through the angiopoietin-Tie system. Nat Rev Mol Cell Biol.

[92] Darland DC, Massingham LJ, Smith SR, Piek E, Saint-Geniez M, D’Amore PA (2003). Pericyte production of cell-associated VEGF is differentiation-dependent and is associated with endothelial survival. Dev Biol.

[93] Park DY, Lee J, Kim J, Kim K, Hong S, Han S, Kubota Y, Augustin HG, Ding L, Kim JW, Kim H, He Y, Adams RH, Koh GY (2017). Plastic roles of pericytes in the blood-retinal barrier. Nat Commun.

[94] Wang S, Zaitoun IS, Johnson RP, Jamali N, Gurel Z, Wintheiser CM, Strasser A, Lindner V, Sheibani N, Sorenson CM (2017). Bim expression in endothelial cells and pericytes is essential for regression of the fetal ocular vasculature. PLoS ONE.

[95] Simonavicius N, Ashenden M, van Weverwijk A, Lax S, Huso DL, Buckley CD, Huijbers IJ, Yarwood H, Isacke CM (2012). Pericytes promote selective vessel regression to regulate vascular patterning. Blood.

[96] Phng LK, Potente M, Leslie JD, Babbage J, Nyqvist D, Lobov I, Ondr JK, Rao S, Lang RA, Thurston G, Gerhardt H (2009). Nrarp coordinates endothelial Notch and Wnt signaling to control vessel density in angiogenesis. Dev Cell.

[97] Baffert F, Le T, Sennino B, Thurston G, Kuo CJ, Hu-Lowe D, McDonald DM (2006). Cellular changes in normal blood capillaries undergoing regression after inhibition of VEGF signaling. Am J Physiol Heart Circ Physiol.

[98] Baluk P, Lee CG, Link H, Ator E, Haskell A, Elias JA, McDonald DM (2004). Regulated angiogenesis and vascular regression in mice overexpressing vascular endothelial growth factor in airways. Am J Pathol.

[99] Ishida S, Yamashiro K, Usui T, Kaji Y, Ogura Y, Hida T, Honda Y, Oguchi Y, Adamis AP (2003). Leukocytes mediate retinal vascular remodeling during development and vaso-obliteration in disease. Nat Med.

[100] Shen J, Xie B, Dong A, Swaim M, Hackett SF, Campochiaro PA (2007). In vivo immunostaining demonstrates macrophages associate with growing and regressing vessels. Invest Ophthalmol Vis Sci.

[101] Fantin A, Vieira JM, Gestri G, Denti L, Schwarz Q, Prykhozhij S, Peri F, Wilson SW, Ruhrberg C (2010). Tissue macrophages act as cellular chaperones for vascular anastomosis downstream of VEGF-mediated endothelial tip cell induction. Blood.

[102] Zhang Y, Xu B, Chen Q, Yan Y, Du J, Du X (2018). Apoptosis of endothelial cells contributes to brain vessel pruning of zebrafish during development. Front Mol Neurosci.

[103] Cheng C, Haasdijk R, Tempel D, van de Kamp EH, Herpers R, Bos F, Den Dekker WK, Blonden LA, de Jong R, Burgisser PE, Chrifi I, Biessen EA, Dimmeler S, Schulte-Merker S, Duckers HJ (2012). Endothelial cell-specific FGD5 involvement in vascular pruning defines neovessel fate in mice. Circulation.

[104] Korn C, Augustin HG (2015). Mechanisms of vessel pruning and regression. Dev Cell.

[105] Kochhan E, Lenard A, Ellertsdottir E, Herwig L, Affolter M, Belting HG, Siekmann AF (2013). Blood flow changes coincide with cellular rearrangements during blood vessel pruning in zebrafish embryos. PLoS ONE.

[106] Wietecha MS, Cerny WL, DiPietro LA (2013). Mechanisms of vessel regression: toward an understanding of the resolution of angiogenesis. Curr Top Microbiol Immunol.

[107] Savant S, La Porta S, Budnik A, Busch K, Hu J, Tisch N, Korn C, Valls AF, Benest AV, Terhardt D, Qu X, Adams RH, Baldwin HS, Ruiz de Almodovar C, Rodewald HR, Augustin HG (2015). The orphan receptor Tie1 controls angiogenesis and vascular remodeling by differentially regulating Tie2 in Tip and stalk cells. Cell reports.

[108] Scholz B, Korn C, Wojtarowicz J, Mogler C, Augustin I, Boutros M, Niehrs C, Augustin HG (2016). Endothelial RSPO3 controls vascular stability and pruning through non-canonical WNT/Ca(2+)/NFAT signaling. Dev Cell.

[109] Franco CA, Jones ML, Bernabeu MO, Vion AC, Barbacena P, Fan J, Mathivet T, Fonseca CG, Ragab A, Yamaguchi TP, Coveney PV, Lang RA, Gerhardt H (2016). Non-canonical Wnt signalling modulates the endothelial shear stress flow sensor in vascular remodelling. Elife.

[110] Hubert KE, Davies MH, Stempel AJ, Griffith TS, Powers MR (2009). TRAIL-deficient mice exhibit delayed regression of retinal neovascularization. Am J Pathol.

[111] Chen Q, Jiang L, Li C, Hu D, Bu JW, Cai D, Du JL (2012). Haemodynamics-driven developmental pruning of brain vasculature in zebrafish. Plos Biol.

[112] Lenard A, Daetwyler S, Betz C, Ellertsdottir E, Belting HG, Huisken J, Affolter M (2015). Endothelial cell self-fusion during vascular pruning. Plos Biol.

[113] Zwerts F, Lupu F, De Vriese A, Pollefeyt S, Moons L, Altura RA, Jiang Y, Maxwell PH, Hill P, Oh H, Rieker C, Collen D, Conway SJ, Conway EM (2007). Lack of endothelial cell survivin causes embryonic defects in angiogenesis, cardiogenesis, and neural tube closure. Blood.

[114] Kang TB, Jeong JS, Yang SH, Kovalenko A, Wallach D (2018). Caspase-8 deficiency in mouse embryos triggers chronic RIPK1-dependent activation of inflammatory genes, independently of RIPK3. Cell Death Differ.

[115] Qiu W, Carson-Walter EB, Liu H, Epperly M, Greenberger JS, Zambetti GP, Zhang L, Yu J (2008). PUMA regulates intestinal progenitor cell radiosensitivity and gastrointestinal syndrome. Cell Stem Cell.

[116] Biancone L, Martino AD, Orlandi V, Conaldi PG, Toniolo A, Camussi G (1997). Development of inflammatory angiogenesis by local stimulation of Fas in vivo. J Exp Med.

[117] Hanggi K, Vasilikos L, Valls AF, Yerbes R, Knop J, Spilgies LM, Rieck K, Misra T, Bertin J, Gough PJ, Schmidt T, de Almodovar CR, Wong WW (2017). RIPK1/RIPK3 promotes vascular permeability to allow tumor cell extravasation independent of its necroptotic function. Cell Death Dis.

[118] Matsumoto T, Turesson I, Book M, Gerwins P, Claesson-Welsh L (2002). p38 MAP kinase negatively regulates endothelial cell survival, proliferation, and differentiation in FGF-2-stimulated angiogenesis. J Cell Biol.

[119] Li JH, Kirkiles-Smith NC, McNiff JM, Pober JS (2003). TRAIL induces apoptosis and inflammatory gene expression in human endothelial cells. J Immunology.

[120] Na HJ, Hwang JY, Lee KS, Choi YK, Choe J, Kim JY, Moon HE, Kim KW, Koh GY, Lee H, Jeoung D, Won MH, Ha KS, Kwon YG, Kim YM (2014). TRAIL negatively regulates VEGF-induced angiogenesis via caspase-8-mediated enzymatic and non-enzymatic functions. Angiogenesis.

[121] Cantarella G, Di Benedetto G, Ribatti D, Saccani-Jotti G, Bernardini R (2014). Involvement of caspase 8 and c-FLIPL in the proangiogenic effects of the tumour necrosis factor-related apoptosis-inducing ligand (TRAIL). FEBS J.

[122] Sosna J, Philipp S, Fuchslocher Chico J, Saggau C, Fritsch J, Foll A, Plenge J, Arenz C, Pinkert T, Kalthoff H, Trauzold A, Schmitz I, Schutze S, Adam D (2016). Differences and similarities in TRAIL- and tumor necrosis factor-mediated necroptotic signaling in cancer cells. Mol Cell Biol.

[123] Vercammen D, Beyaert R, Denecker G, Goossens V, Van Loo G, Declercq W, Grooten J, Fiers W, Vandenabeele P (1998). Inhibition of caspases increases the sensitivity of L929 cells to necrosis mediated by tumor necrosis factor. J Exp Med.

[124] Kearney CJ, Martin SJ (2017). An inflammatory perspective on necroptosis. Mol Cell.

[125] Richardson BC, Lalwani ND, Johnson KJ, Marks RM (1994). Fas ligation triggers apoptosis in macrophages but not endothelial cells. Eur J Immunol.

[126] Sata M, Suhara T, Walsh K (2000). Vascular endothelial cells and smooth muscle cells differ in expression of Fas and Fas ligand and in sensitivity to Fas ligand-induced cell death: implications for vascular disease and therapy. Arterioscler Thromb Vasc Biol.

[127] Walsh K, Sata M (1999). Negative regulation of inflammation by Fas ligand expression on the vascular endothelium. Trends Cardiovasc Med.

[128] Smyth LA, Brady HJ (2005). cMet and Fas receptor interaction inhibits death-inducing signaling complex formation in endothelial cells. Hypertension.

[129] Takemura Y, Fukuo K, Yasuda O, Inoue T, Inomata N, Yokoi T, Kawamoto H, Suhara T, Ogihara T (2004). Fas signaling induces Akt activation and upregulation of endothelial nitric oxide synthase expression. Hypertension.

[130] Shioiri T, Muroi M, Hatao F, Nishida M, Ogawa T, Mimura Y, Seto Y, Kaminishi M, Tanamoto K (2009). Caspase-3 is activated and rapidly released from human umbilical vein endothelial cells in response to lipopolysaccharide. Biochim Biophys Acta.

[131] Suresh K, Carino K, Johnston L, Servinsky L, Machamer CE, Kolb TM, Lam H, Dudek SM, An SS, Rane MJ, Shimoda LA, Damarla M (2019). A nonapoptotic endothelial barrier-protective role for caspase-3. Am J Physiol Lung Cell Mol Physiol.

[132] Kondo S, Tang Y, Scheef EA, Sheibani N, Sorenson CM (2008). Attenuation of retinal endothelial cell migration and capillary morphogenesis in the absence of bcl-2. Am J Physiol Cell Physiol.

[133] Scott A, Fruttiger M (2010). Oxygen-induced retinopathy: a model for vascular pathology in the retina. Eye (Lond).

[134] Kim CB, D’Amore PA, Connor KM (2016). Revisiting the mouse model of oxygen-induced retinopathy. Eye Brain.

[135] Sapieha P, Hamel D, Shao Z, Rivera JC, Zaniolo K, Joyal JS, Chemtob S (2010). Proliferative retinopathies: angiogenesis that blinds. Int J Biochem Cell B.

[136] Donahue ML, Phelps DL, Watkins RH, LoMonaco MB, Horowitz S (1996). Retinal vascular endothelial growth factor (VEGF) mRNA expression is altered in relation to neovascularization in oxygen induced retinopathy. Curr Eye Res.

[137] Shih SC, Ju M, Liu N, Smith LE (2003). Selective stimulation of VEGFR-1 prevents oxygen-induced retinal vascular degeneration in retinopathy of prematurity. J Clin Invest.

[138] Lofqvist C, Chen J, Connor KM, Smith AC, Aderman CM, Liu N, Pintar JE, Ludwig T, Hellstrom A, Smith LE (2007). IGFBP3 suppresses retinopathy through suppression of oxygen-induced vessel loss and promotion of vascular regrowth. Proc Natl Acad Sci USA.

[139] Hartnett ME (2015). Pathophysiology and mechanisms of severe retinopathy of prematurity. Ophthalmology.

[140] Mizutani M, Kern TS, Lorenzi M (1996). Accelerated death of retinal microvascular cells in human and experimental diabetic retinopathy. J Clin Invest.

[141] Hammes HP, Feng Y, Pfister F, Brownlee M (2011). Diabetic retinopathy: targeting vasoregression. Diabetes.

[142] Zaitoun IS, Johnson RP, Jamali N, Almomani R, Wang S, Sheibani N, Sorenson CM (2015). Endothelium expression of Bcl-2 is essential for normal and pathological ocular vascularization. PLoS ONE.

[143] Grant ZL, Whitehead L, Wong VHY, He Z, Yan RY, Miles AR, Benest AV, Bates DO, Prahst C, Bentley K, Bui BV, Symons RC, Coultas L (2020). Blocking endothelial apoptosis revascularises the retina in a model of ischemic retinopathy. J Clin Invest.

[144] Barreiro R, Schadlu R, Herndon J, Kaplan HJ, Ferguson TA (2003). The role of Fas-FasL in the development and treatment of ischemic retinopathy. Invest Ophthalmol Vis Sci.

[145] Davies MH, Eubanks JP, Powers MR (2003). Increased retinal neovascularization in Fas ligand-deficient mice. Invest Ophthalmol Vis Sci.

[146] Bergers G, Benjamin LE (2003). Tumorigenesis and the angiogenic switch. Nat Rev Cancer.

[147] Folkman J (1971). Tumor angiogenesis: therapeutic implications. N Engl J Med.

[148] Benjamin LE, Golijanin D, Itin A, Pode D, Keshet E (1999). Selective ablation of immature blood vessels in established human tumors follows vascular endothelial growth factor withdrawal. J Clin Invest.

[149] Carmeliet P, Jain RK (2011). Principles and mechanisms of vessel normalization for cancer and other angiogenic diseases. Nat Rev Drug Discov.

[150] Lemke J, von Karstedt S, Zinngrebe J, Walczak H (2014). Getting TRAIL back on track for cancer therapy. Cell Death Differ.

[151] Fox JL, MacFarlane M (2016). Targeting cell death signalling in cancer: minimising ‘Collateral damage’. Br J Cancer.

[152] Shojaei F, Wu X, Malik AK, Zhong C, Baldwin ME, Schanz S, Fuh G, Gerber HP, Ferrara N (2007). Tumor refractoriness to anti-VEGF treatment is mediated by CD11b+Gr1+ myeloid cells. Nat Biotechnol.

[153] Yang L, Joseph S, Sun T, Hoffmann J, Thevissen S, Offermanns S, Strilic B (2019). TAK1 regulates endothelial cell necroptosis and tumor metastasis. Cell Death Differ.

[154] Strilic B, Yang L, Albarran-Juarez J, Wachsmuth L, Han K, Muller UC, Pasparakis M, Offermanns S (2016). Tumour-cell-induced endothelial cell necroptosis via death receptor 6 promotes metastasis. Nature.

